# Appropriate cut-off value for follicle-stimulating hormone in azoospermia to predict spermatogenesis

**DOI:** 10.1186/1477-7827-8-108

**Published:** 2010-09-08

**Authors:** Shyh-Chyan Chen, Ju-Ton Hsieh, Hong-Jeng Yu, Hong-Chiang Chang

**Affiliations:** 1 Department of Urology, National Taiwan University Hospital and National Taiwan University, College of Medicine, Taipei, Taiwan

## Abstract

**Background:**

This study was undertaken to determine the optimal cut-off value for FSH to predict the presence of spermatogenesis in patients with non-obstructive azoospermia.

**Methods:**

A total of 206 non-obstructive azoospermic men were enrolled in this prospective study. By using receiver operating characteristic (ROC) curves, we determined the optimal cut-off value for FSH and evaluated whether the test could adequately predict successful sperm retrieval.

**Results:**

There were 108 non-obstructive azoospermic patients who had evidence of spermatogenesis (group A) and achieved success in sperm retrieval. Another 98 non-obstructive azoospermic patients (group B) failed in sperm retrieval. The mean value of serum FSH in group B was significantly higher than in group A (28.03 +/- 14.56 mIU/mL vs 7.94 +/- 4.95 mIU/mL, p < 0.01; respectively). The area under the receiver operating characteristic curves were 0.939 +/- 0.02 and a cut-off value of 19.4 mIU/mL discriminated between group A and B with a sensitivity of 70%. The positive predictive value for failed sperm retrieval (group B) can reach 100%.

**Conclusions:**

Elevated plasma levels of FSH of more than 19.4 mIU/mL could be used as a reliable criterion for a trial of sperm retrieval from testes in artificial reproductive techniques.

## Background

A normal hypothalamic-pituitary-gonadal axis is usually a necessary prerequisite for spermatogenesis[1]. Hormonal control of the hypothalamic-pituitary-gonadal axis involves a negative feedback mechanism[2,3]. The gonadotropin-releasing hormone (GnRH) is secreted from the hypothalamus and stimulates the production of follicle-stimulating hormone (FSH) and luteinizing hormone (LH) from the basophilic cells in the anterior lobe of the pituitary gland[4]. The primary site of FSH action is on the Sertoli cells of the seminiferous tubules to maintain normal spermatogenesis. The Sertoli cells interact with the spermatogenic cells and produce the inhibin B which suppresses FSH secretion from the pituitary gland. In the condition of spermatogenic failure, the production of inhibin B is decreased and plasma FSH increases[5].

Elevated FSH levels have been shown to be associated with damage to the germinal epithelium of the seminiferous tubules[6]. Plasma FSH levels usually correlate inversely with spermatogenesis, and therefore FSH is considered to be a clinically useful endocrine marker in the evaluation of infertile men. There is a consensus that men with azoospermia and testicular atrophy with significantly elevated FSH levels should undergo testicular biopsy if *in vitro *fertilization with intracytoplasmic sperm injection (ICSI) is an acceptable approach for both partners[7]. However, the diagnostic criterion of elevated plasma FSH level is still controversial and conflicting recommendations with regard to FSH level have been proposed [8,9]. Because an artificial reproductive technique is an expensive procedure, we need an optimal cut-off level for FSH to predict the success of sperm retrieval for infertile couples. There is considerable overlap of increased FSH levels and completeness of spermatogenesis so that even patients with Sertoli-cell-only syndrome might have normal FSH levels and conversely, patients with highly elevated FSH levels do not necessarily lose spermatogenesis. Although two to three times the normal value has been recommended, an optimal cut-off value in the differential diagnostic evaluation of spermatogenic failure and obstructive azoospermia has not yet been determined [8,9]. This study was undertaken to determine the optimal cut-off value for FSH to predict the presence of spermatogenesis in non-obstructive azoospermic patients.

## Methods

### Patients

From April 2003 to December 2006, a total of 206 non-obstructive azoospermic men were enrolled in this prospective study. The hospital ethical committee approved the study protocol, and written informed consent was obtained from each patient. These patients underwent a complete examination to determine the etiology of azoospermia, including a detailed history, physical examination, at least three consecutive semen analyses, chromosomal karyotyping study and endocrinology profile (FSH, LH, prolactin, and testosterone). All semen samples were obtained by masturbation after 3 days of sexual abstinence and were evaluated on at least three separate occasions, separated by a 3-week interval. After liquefaction at room temperature, semen volume, pH, sperm concentration, motility and morphology were determined following WHO guidelines for semen analysis[10]. Follicle-stimulating hormone was determined by chemiluminescent immunometric assay using commercial kits (IMMULITE 2000 FSH, Deerfield, IL USA). To differentiate between obstructive and non-obstructive azoospermia, a comprehensive diagnostic and therapeutic procedure was carried out in one operative session[11]. Briefly, wet prep analysis of testis tissue was performed to detect the presence of sperm[12]. If spermatogenesis was noted, then vasography with saline infusion was performed to make sure the vas deferens was patent. Either sperm retrieval for freezing or microscopic vasoepididymostomy can be performed. These azoospermia cases were defined as successful sperm retrieval (group A). If sperm were not found by both wet prep examination and microscopic testicular sperm extract, then ordinary testicular biopsy was performed. At least three pieces of specimen for each testis will be collected and these specimens were fixed in Bouin's solution for final histological diagnosis. These cases, in which there were failure of sperm retrieval and histological spermatogenic defects, such as Sertoli-cell-only, maturation arrests, or severe fibrosis, were defined as spermatogenic failure (group B).

### Statistical analysis

All results are expressed as mean±standard deviation (S.D.). The Student *t *test was used to compare serum FSH levels between groups A and B. *P *values less than 0.05 were considered significant. Receiver operating characteristic (ROC) curves were used to determine the optimal cut-off value for FSH to assess whether the test could adequately discriminate between groups A and B. The ability of serum FSH to predict sperm retrieval was estimated on the basis of sensitivity and specificity at various cut-off values. An area under the ROC curve of 1.0 indicates perfect discrimination, whereas an area of 0.5 indicates that the test discriminates no better than chance[13].

## Results

The characteristics of the patients are presented in Table 1. No significant difference was observed in mean age between the two groups. There were 108 non-obstructive azoospermic patients who had evidence of spermatogenesis and success in sperm retrieval (group A). The other 96 azoospermic patients had spermatogenic failure (group B) and failed in sperm retrieval. The histological findings in testicular biopsies were spermatogenic defects such as hypospermatogenesis, maturation arrest, and Sertoli-cell-only syndrome. As shown in Table 1, the mean value of serum FSH in group B (28.03 ± 14.56 mIU/mL) was significantly higher than that in group A (7.94 ± 4.95 mIU/mL, *p *< 0.01). In order to confirm the ability of an elevated FSH level to discriminate between these two groups we subjected our results to receiver operating characteristic (ROC) curve analysis.

**Table 1:** Patient characteristics and follicle-stimulating hormone (FSH) values.

Sperm retrieval	Successful (*n *= 108)	Failed (*n *= 98)	
Age (years)			
Mean ± SD	30.1 ± 4.2	29.8 ± 4.5	*p *= 0.69
Range	24-38	22-36	
FSH (mIU/mL)			
Mean ± SD	7.94 ± 4.95	28.03 ± 14.56	*p *< 0.01
Range	1.8-19.0	2.2-75.3	

**Figure 1:**
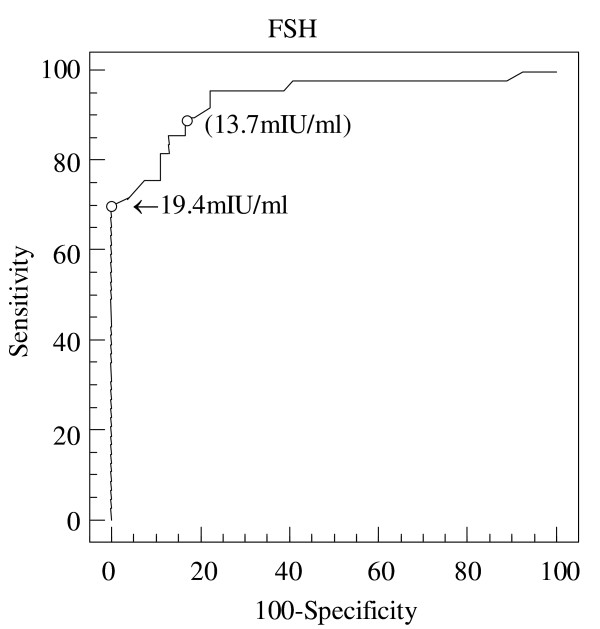
**Depiction of ROC curves of serum FSH can discriminate between successful or failed sperm retrieval**. Depiction of receiver operating characteristic (ROC) curves of serum follicle-stimulating hormone (FSH) can discriminate between successful or failed sperm retrieval. The area under the ROC curves was 0.939 ± 0.02 (with a 95% confidence interval for the area being between 0.891 and 0.986). A FSH cut-off point of 13.7 mIU/mL discriminated between groups A and B with a sensitivity of 85.7% and a specificity of 87.0%. When the cut-off point was increased to 19.4 mIU/mL, the sensitivity of FSH level was 70% and the specificity was 100%. Arrows indicate the cut-off point for serum FSH 19.4 mIU/mL.

**Figure 2:**
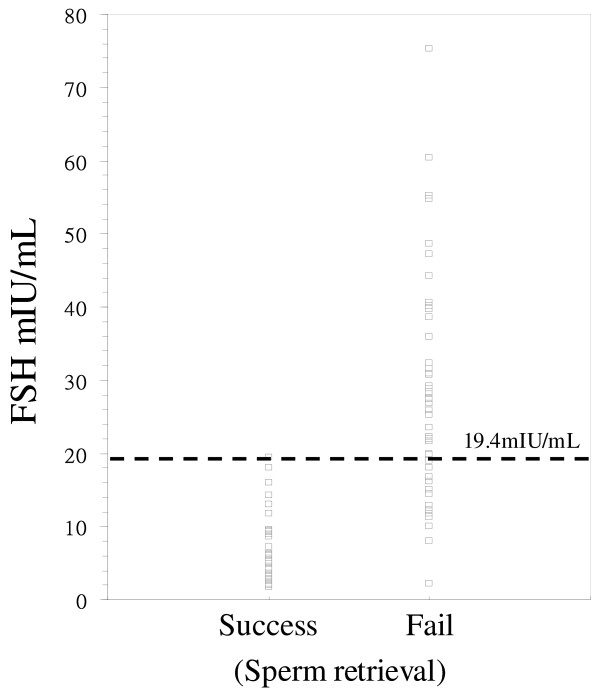
**Dot diagram shows the serum FSH data and the cut-off point for sperm retrieval**. Dot diagram shows the serum follicle-stimulating hormone data and the cut-off point for sperm retrieval. The best cut-off point for failed sperm retrieval in non-obstructive azoospermic patients is 19.4 mIU/mL. However, a value of FSH lower than 19.4 mIU/mL does not predict normal spermatogenesis and successful sperm retrieval.

Figure 1 depicts the ROC curves for FSH concentration. The area under the ROC curves was 0.939 ± 0.02 (with a 95% CI for the area being between 0.891 and 0.986). A cut-off value of 13.7 mIU/mL discriminated between groups A and B with a sensitivity of 85.7% and a specificity of 87.0%. When the cut-off value was increased to 19.4 mIU/mL, the sensitivity was 85.7% and the positive predictive value could reach 100%. As shown in Figure 2, a cut-off value of 19.4 mIU/mL discriminated between groups A and group B with a sensitivity of 85.7% and a specificity of 100%. The positive predictive value for failed sperm retrieval (group B) can reach 100%.

## Discussion

The etiology of hypergonadotropism in azoospermic patients has been well studied and is known to involve a negative feedback mechanism involving Sertoli cells and inhibin B[14]. Serum FSH can increase to as high as two to four fold the normal range. Although FSH can facilitate the initiation of spermatogenesis in pubertal males, hypogonadal men still can demonstrate fertility in the absence of FSH. Only the combination of FSH and testosterone can support a qualitatively and quantitatively full normal level of spermatogenesis.

In patients with obstructive azoospermia, if reconstructive surgery fails or is not feasible, microscopic epididymal sperm aspiration (MESA) or testicular sperm extraction (TESE) is the method of choice for recovering spermatozoa for *in vitro *fertilization (IVF). In patients with non-obstructive azoospermia, TESE is the method of choice for recovering spermatozoa as a male therapeutic approach in ICSI. Due to the psychological, economic, or physical burden of the IVF procedure, useful criteria to predict the absence of spermatozoa are highly important for making a decision of sperm retrieval procedure. Although histopathology has been found to be the best method for predicting successful sperm recovery in non-obstructive azoospermic patients, this method requires an operative procedure[5,15]. Several non-invasive laboratory tools have been suggested for prediction of the presence of spermatozoa, such as the levels of inhibin B and FSH in serum. Inhibin B level is a parameter that has been shown to be associated with maturation arrest; however, it is not used routinely and is not diagnostic[16,17]. The combination of inhibin B and FSH levels has been reported to be a better predictor for the presence of sperm in TESE. However, the prediction is not absolutely reliable[18]. FSH has been used as a marker of spermatogenesis, but the optimal criterion for serum level has still not been precisely determined[5]. A two-fold increase above the upper limit of normal serum FSH level has usually been used as the cut-off value for spermatogenesis, but this does not provide a better predictive rate than histopathology[19]. It has been reported that when elevated plasma levels of FSH are three fold above the normal limit (more than 27 mIU/mL) this precludes, with a probability of 95%, the existence of full spermatogenesis[20]. However, with regard to FSH plasma levels, a high FSH level can not be used for the diagnosis of spermatogenic maturation arrest and Sertoli-cell-only syndrome[20].

The predictive power for detecting oligospermia among men with FSH above 10 IU/L has been found to be 100%[5]. In a study of 106 azoospermic patients, small testicular size (< 4 cm) and elevated FSH (> 10) was associated with a decrease in the success rate of sperm retrieval rate from 77% to 29%[21]. In another report it was found that a level above 60 mIU/mL of serum FSH could completely exclude the possibility of sperm retrieval from testis; the sperm retrieval rate was 100% for FSH ≤15 mIU/mL [22]. Although measurement of serum FSH is a non-invasive test for predicting success rate of sperm retrieval in azoospermic patients, the cut-off value for FSH for predicting spermatogenesis is rather variable. We suggest that one major reason for the variability is due to the development of microsurgical testicular sperm retrieval (microTESE). The microTESE has better sperm retrieval rate than random testicular biopsy. Many men who have been diagnosed with non-obstructive azoospermia either due to the pathological conditions known as hypospermatogenesis or Sertoli-cell only syndrome have been found to have foci of sperm production within the testicles. This observation has revolutionized an old concept that the testis produced sperm in a uniform fashion and has lead to the new concept of focal spermatogenesis. In our study, we found that a low FSH level (< 19.4 mIU/mL) is not a predictive criterion for success sperm retrieval. A comprehensive diagnostic evaluation and reconstructive operative procedure are indicated for azoospermic men with a low FSH level (< 19.4 mIU/mL). But men with elevated FSH (> 19.4 mIU/mL) would benefit from a cut-off value for FSH to predict the failure for sperm retrieval. This is very important for microTESE, because fresh testicular sperm will be needed to approve patients for successful concomitant IVF and ICSI procedures.

## Conclusions

Our data suggest that plasma FSH levels can be used to predict the failure of sperm retrieval in patients with azoospermia. Elevated plasma levels of FSH (> 19.4 mIU/mL) can preclude the presence of spermatogenesis with a probability of 100%. When FSH was above the level of 19.4 mIU/mL, there were no successful sperm retrievals in our series. However, a normal value of FSH does not predict normal spermatogenesis and successful sperm retrieval. We suggest that a cut-off value of FSH could be used as a reliable criterion for selecting patients for a trial of sperm retrieval from testes using an artificial reproductive technique.

## Competing interests

The authors declare that they have no competing interests.

## Authors' contributions

JTH, SCC an HJY have made substantial contributions to conception and design, and performed the statistical analysis. HCC participated in its design, in drafting the manuscript and revising it for important intellectual content. All authors read and approved the final manuscript.
